# Epigallocatechin-3-Gallate (EGCG), an Active Compound of Green Tea Attenuates Acute Lung Injury Regulating Macrophage Polarization and Krüpple-Like-Factor 4 (KLF4) Expression

**DOI:** 10.3390/molecules25122853

**Published:** 2020-06-20

**Authors:** Saleh A. Almatroodi, Ahmad Almatroudi, Mohammed A. Alsahli, Mohammad A. Aljasir, Mansoor Ali Syed, Arshad Husain Rahmani

**Affiliations:** 1 Department of Medical Laboratories, College of Applied Medical Sciences, Qassim University, Buraydah 52571, Saudi Arabia; smtrody@qu.edu.sa (S.A.A.); aamtrody@qu.edu.sa (A.A.); shly@qu.edu.sa (M.A.A.); mjasr@qu.edu.sa (M.A.A.); 2 Translational Research Lab, Department of Biotechnology, Faculty of Natural Sciences, Jamia Millia Islamia, New Delhi 110025, India; smansoor@jmi.ac.in

**Keywords:** epigallocatechin-3-gallate (EGCG), macrophage polarisation, acute lung injury

## Abstract

Acute lung injury (ALI)/acute respiratory distress syndrome (ARDS) are serious clinical complications with a high frequency of morbidity and mortality. The initiation and amplification of inflammation is a well-known aspect in the pathogenesis of ALI and related disorders. Therefore, inhibition of the inflammatory mediators could be an ideal approach to prevent ALI. Epigallocatechin-3-gallate (EGCG), a major constituent of green tea, has been shown to have protective effects on oxidative damage and anti-inflammation. The goal of the present study was to determine whether EGCG improves phenotype and macrophage polarisation in LPS-induced ALI. C57BL/6 mice were given two doses of EGCG (15 mg/kg) intraperitoneally (IP) 1 h before and 3 h after LPS instillation (2 mg/kg). EGCG treatment improved histopathological lesions, Total Leucocyte count (TLC), neutrophils infiltration, wet/dry ratio, total proteins and myeloperoxidase (MPO) activity in LPS-induced lung injury. The results displayed that EGCG reduced LPS-induced ALI as it modulates macrophage polarisation towards M2 status. Furthermore, EGCG also reduced the expression of proinflammatory M1 mediators iNOS TNF-α, IL-1β and IL-6 in the LPS administered lung microenvironment. In addition, it increased the expression of KLF4, Arg1 and ym1, known to augment the M2 phenotype of macrophages. EGCG also alleviated the expression of 8-OHdG, nitrotyrosine, showing its ability to inhibit oxidative damage. TREM1 in the lung tissue and improved lung regenerative capacity by enhancing Ki67, PCNA and Ang-1 protein expression. Together, these results proposed the protective properties of EGCG against LPS-induced ALI in may be attributed to the suppression of M1/M2 macrophages subtype ratio, KLF4 augmentation, lung cell regeneration and regulating oxidative damage in the LPS-induced murine ALI.

## 1. Introduction

Acute lung injury (ALI)/acute respiratory distress syndrome (ARDS) are common serious diseases that may lead to death [1]. Its main pathological features are lung oedema, reduced lung function, and disbalance of the gas exchange [1]. ALI can be persuaded by several direct or indirect insults of the lung [2,3], but the major cause is infection, which is characterised by widespread lung inflammation [3,4]. Upon lipopolysaccharide (LPS) stimulation, TLR4 is recruited and production of cytokines, which triggers inflammation and finally acute lung injury [5]. Despite the substantial advancement in reducing acute lung injury, the frequency and deaths of ALI are still high. Therefore, it is critical to search for effective therapeutics to manage ALI.

Macrophages demonstrate extraordinary plasticity that permits them to modify their phenotype and competently respond to external stimuli [6,7]. Researchers have developed a model system which divides macrophages into M1 phenotype-the proinflammatory state and M2 phenotype represent the anti-inflammatory state [6,8]. The classification of M1 and M2 phenotypes of macrophages has provided an important tool for the understanding of inflammatory progression. The molecular mechanisms of regulating M1/M2 polarisation remain to be elucidated.

Krüpple-like factor-4 (KLF4) is a transcription factor, belongs to the KLF family of proteins which contain zinc-finger DNA binding domain and regulates various cell processes [9,10]. KLF4 performs numerous physiological functions and is involved in several disease processes [11,12,13]. Recent studies have shown that KLF4 expression is decreased in lung injury and pulmonary arterial hypertension [14,15]. KLF4 is expressed in myeloid cells and known for its role in macrophage polarisation and functions [13,14].

**Scheme 1:**
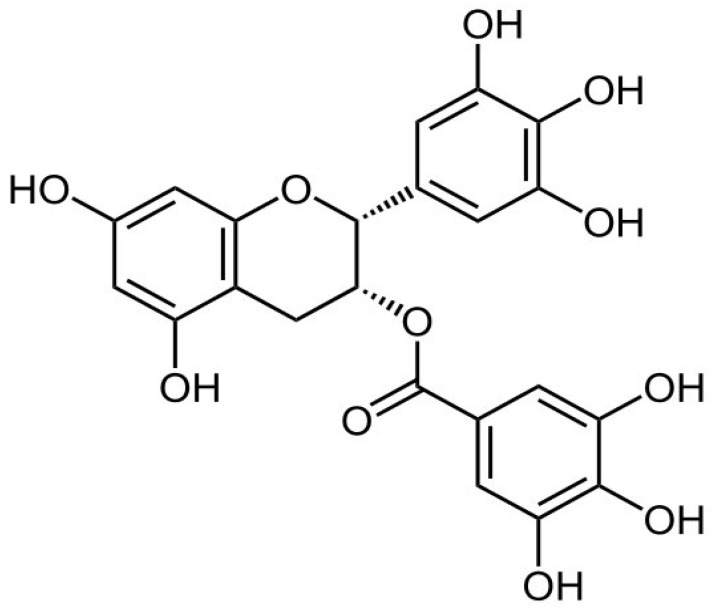
Chemical structure of epigallocatechin-3-gallate (EGCG), the major component of green tea.

Epigallocatechin-3-gallate (EGCG) is one of the major components of green tea [16] (Scheme 1). Several former studies have stated that EGCG has encouraging effects in mitigation of inflammation and lung injury [17,18]. Cumulative studies illustrate that EGCG prevents inflammation suppressing TLR signalling and augmenting antioxidant defence [19].

While numerous studies have evaluated the role of natural compounds on KLF4, the role of EGCG on macrophage polarisation and KLF4 expression has not been elucidated. Thus, we hypothesised that EGCG modulates KLF4, which may control macrophage polarisation, oxidative stress, cell proliferation and ALI. We demonstrate that treatment of macrophages with EGCG inhibits LPS-induced inflammatory and M1 polarisation markers and EGCG augmented IL-4-induced M2 phenotype markers. We also found that EGCG induces KLF4 expression which is a regulator of M2 macrophage phenotype. We also demonstrate that EGCG effectively reduced LPS-induced lung injury inhibiting M1 polarisation and inflammatory mediators and increasing M2 markers including KLF4. Furthermore, we showed reduced oxidative stress and enhanced cell proliferation in EGCG + LPS-treated mice lungs. Thus, both in vitro and in vivo experiments show that EGCG plays a critical role in M2 polarisation and KLF4 expression.

## 2. Results

### 2.1. EGCG Treatment Improves LPS-Induced Acute Lung Injury

**Figure 1:**
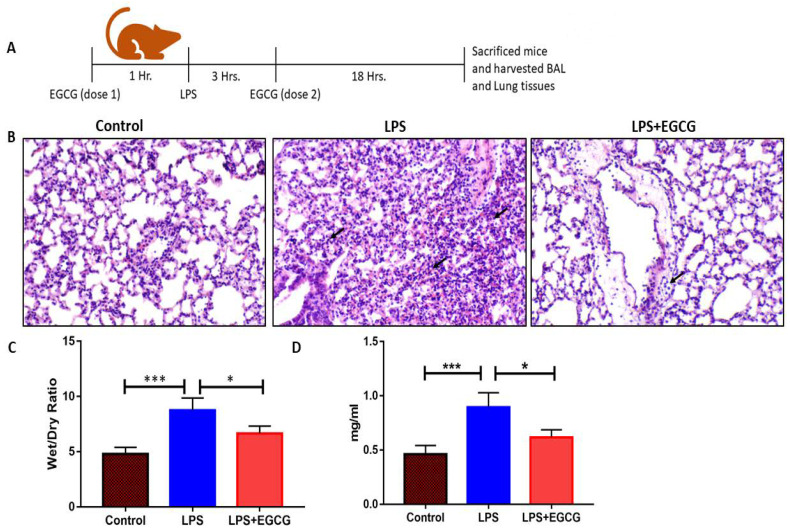
EGCG reduced histopathological damage and oedema in lipopolysaccharide (LPS)(IT)-induced lung. (**A**) Schematic to perform mice experiments for the study. (**B**) Representative histological slides of the lungs of designated groups (H&E staining, 10×). Enhanced inflammatory cells infiltration in the alveolar area of the lung in the LPS-instilled group when compared with the control group. Administration of EGCG suppressed infiltration of cells and less dense histoarchitecture of lung tissue. (**C**) Wet/dry ratio was significantly enhanced after LPS instillation, administration of EGCG significantly inhibits LPS-induced lung wet/dry ratio. (**D**) Total protein of bronchoalveolar lavage (BAL) fluid was measured after 18 h of acute lung injury (ALI) induction in different treatment groups. Significant upregulation in BAL protein levels after LPS(IT) instillation, treatment of EGCG suppressed this increased protein levels. Significant difference * *p* < 0.05 and *** *p* < 0.001.

**Figure 2:**
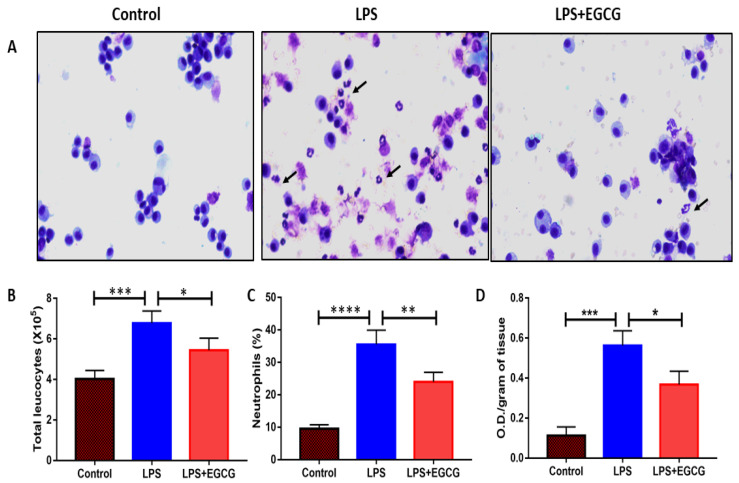
Effects of EGCG on total leukocyte count, and neutrophil differential count and myeloperoxidase (MPO) activity in the BAL of LPS-induced ALI mice. Total BAL fluid was collected after 18 h of ALI instillation from different groups. (**A**) Representative slides show infiltrated cells, cytospin was performed and stained with HEMA of different groups. Enhanced polymorphonuclear neutrophils in the cytospin slides of the LPS-instilled group with control and EGCG treatment groups. (**B**) The LPS group showed a significant increase in the TLC compared with the control and the treatment with EGCG reversed this effect. (**C**) The neutrophil cells percent had a significant enhancement in the LPS(IT) group compared with the control group and the EGCG treatment reduced significantly the percent of neutrophils. (**D**) The lung MPO activity showed a significant rise in the LPS-instilled group compared with the control group and the EGCG administration reduced LPS-induced BAL MPO levels. Significant difference * *p* < 0.05, ** *p* < 0.01, *** *p* < 0.001 and **** *p* < 0.0001. Data represent the mean ± SEM (*n* = 4 animals per group).

The 15 mg/kg EGCG was administered IP injection for twice and 50 μL of LPS (2 mg/kg) or PBS were-instilled intratracheally to induce acute lung injury (Figure 1A). The effects of EGCG treatment on LPS-induced lung histopathological damage were analysed. As expected, an obvious lung pathological damage, including oedema, large infiltration of inflammatory cells, destruction of tracheal and alveolar cells was detected after 18 h of LPS instillation (Figure 1B). EGCG treatment noticeably reduced LPS-induced histopathological damages in the lung (Figure 1B). As shown in Figure 1C, the level of albumin in BLAF was markedly increased 18 h after LPS administration. Similarly, the level of lung wet/dry ratio was also elevated after LPS exposure (Figure 1D). Interestingly, the levels of lung wet/dry ratio and BAL albumin were improved after LPS administration (Figure 1C,D). The LPS treatment persuaded inflammatory cell infiltration to BALF, and EGCG administration reduced this nitration effect (Figure 2A). The total cell count and neutrophils differential count were increased significantly in the LPS-instilled group; however, EGCG treatment inhibited the TLC as well as neutrophils count in BALF (Figure 2B,C). We also found increased BAL myeloperoxidase activity after LPS instillation, which will be improved after simultaneous administration of EGCG (Figure 2D). There were no significant changes in the BAL macrophage count (data not shown).

### 2.2. EGCG Administration Reduced Inflammatory M1/M2 Macrophage Polarisation

**Figure 3:**
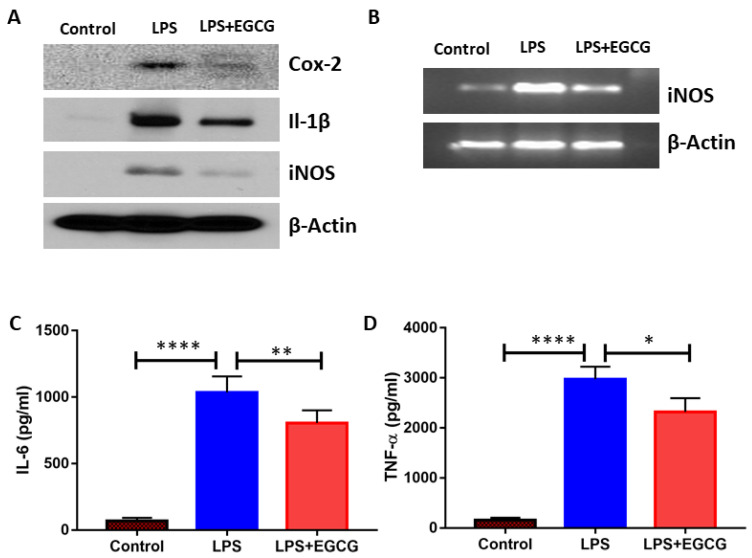
Effects of EGCG on LPS-induced M1 inflammatory mediators. (**A**) RAW cells were stimulated with LPS in the presence or absence of EGCG for 24 h and proinflammatory markers were analysed. Immunoblots of COX2, iNOS and iL-1β demonstrated suppressed activity of these proteins by EGCG in macrophages. (**B**) iNOS mRNAs were measured using RT-PCR (**C**) The BAL TNF-α secretion increased in the LPS-instilled group and declined after EGCG treatment. (**D**) The LPS also increased IL-6 secretion and the EGCG treatment controlled the LPS-induced secretion. Significant difference * *p* < 0.05, ** *p* < 0.01 and **** *p* < 0.0001 The data are expressed as mean ± SEM. (*n* = 4 or more animals per group).

**Figure 4:**
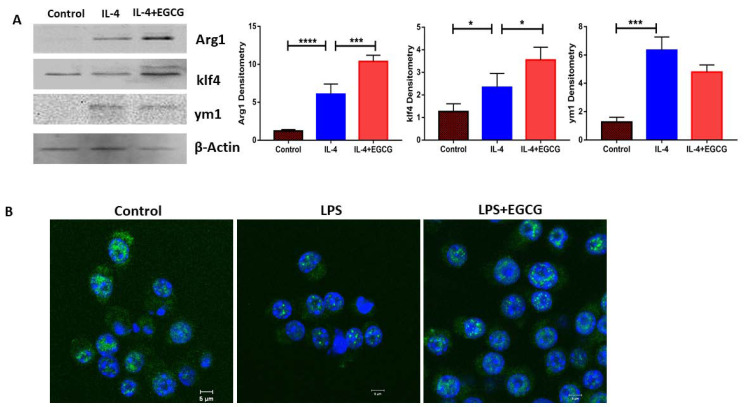
Effects of EGCG on IL-4-induced M2 polarisation. (**A**) RAW cells were stimulated with IL-4 in the presence or absence of EGCG for 24 h and M2 markers were analysed. Immunoblots of Arg1, KLF4 and ym1 demonstrated enhanced expression of these proteins by EGCG in macrophages. (**B**) KLF4 immunofluorescence was demonstrated LPS-induced downregulation, which was enhanced after EGCG incorporation (*n* = 3 or more). Significant difference * *p* < 0.05, *** *p* < 0.001 and **** *p* < 0.0001.

**Figure 5:**
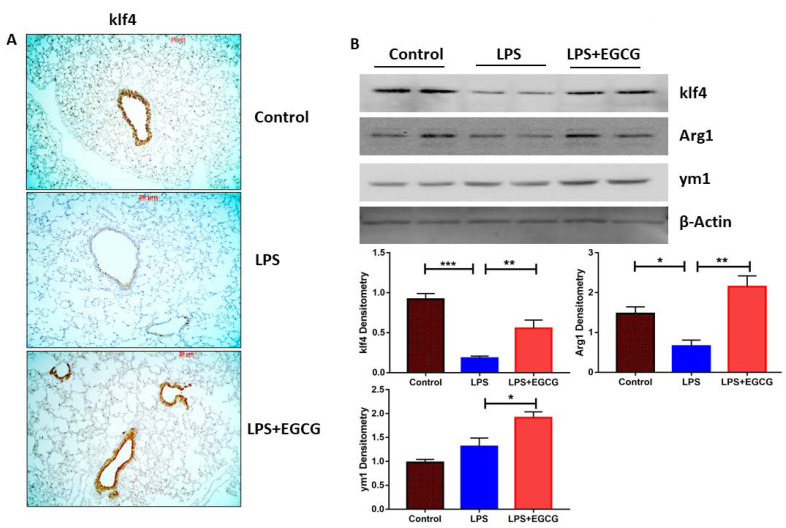
EGCG-induced lung microenvironment towards M2 in LPS-induced ALI. (**A**) The lung KLF4 protein expression shown by immunohistochemistry in the different study groups (10× objective). EGCG treatment caused enhanced expression of lung KLF4 in LPS(IT)-induced ALI mice. (**B**) Western blot of different lung injury groups was performed to evaluate M2 polarisation markers including Arg1, ym1 and KLF4. Actin was used in parallel as internal control. Significant difference * *p* < 0.05, ** *p* < 0.01 and *** *p* < 0.001

Macrophages are polarised into two phenotypes, specifically, classically activated (M1) inflammatory phenotype and alternatively activated (M2) anti-inflammatory cells. LPS is well-known to polarise macrophages toward M1 inflammatory phenotype and reducing M2 phenotype [6]. So, we investigated whether EGCG affects LPS-induced polarisation of macrophages into the inflammatory M1 phenotype in lung macrophages. Our experiments found that EGCG treatment reduced LPS-induced M1 proinflammatory markers, including iNOS, Il-1β and Cox-2 in RAW264.7 (Figure 3A). EGCG treatment also effectively decreased LPS-induced iNOS at the mRNA level in macrophages (Figure 3B). LPS treatment additionally directed to enhance secretion of IL-6 and TNFα, which are well capable of converting lung microenvironment towards M1. Injection of EGCG significantly reduced the concentration of these cytokines (Figure 3C,D), helping to achieve a normal lung microenvironment. We then examined the effect of EGCG on IL-4-induced M2 polarisation markers in RAW macrophages. IL-4 treatment led to augmented expression of M2 marker, specifically Arg-1 and ym-1, which were further increased after EGCG treatment (Figure 4A). KLF4 is a well-known transcriptional regulator of macrophage polarisation and polarise macrophages toward M2 phenotype [13]. So, next, we examined the expressions of KLF4 in macrophages and lung tissues of LPS- and/or EGCG-treated mice. We found enhanced expression of KLF4 in EGCG + IL-4-treated group as compared to IL-4 alone treatment (Figure 4A). A similar trend in KLF4 expression was also observed after immunofluorescence staining of RAW macrophages (Figure 4B). Consistent with the results obtained using RAW cells, we obtained the expression of KLF4 in the LPS-induced ALI was diminished in lung immunohistochemistry, but the treatment of EGCG significantly enhanced expression of KLF4 in lung tissues (Figure 5A). As expected, immunoblots of other M2 markers like Arg1 and ym1, along with KLF4, were also improved in EGCG-treated lung tissues as compared to the LPS-instilled group (Figure 5B). Here, we can depict that EGCG can modulate macrophage polarisation enhancing the expression of KLF4.

### 2.3. EGCG Controls Oxidative Stress Markers 8-OHdG and Nitrotyrosine in Mice Lungs

**Figure 6:**
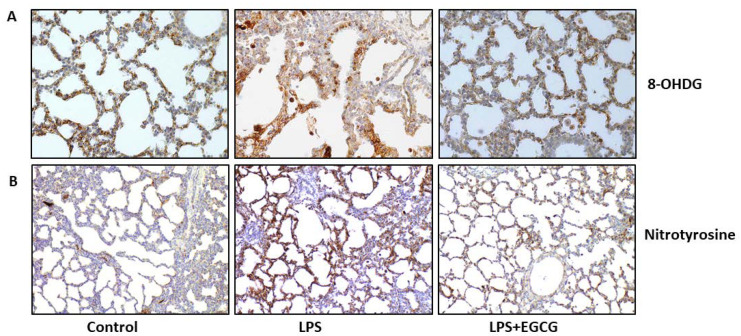
EGCG alleviated oxidative stress in LPS-induced ALI. (**A**) The lung 8-OHDG protein expression shown by immunohistochemistry in the different study groups (10× objective). EGCG treatment caused reduced expression of lung 8-OHDG in LPS(IT)-induced ALI mice. (**B**) Immunostaining of nitrotyrosine showed intense staining in alveolar epithelial cells, LPS(IT) group lung showed enhanced nitrotyrosine staining, although EGCG treatment lessened the expression of nitrotyrosine.

8-hydroxydeoxyguanosine (8-OHdG) is one of the most commonly used markers of oxidative damage [20]. In this study, the results showed that LPS significantly elevated staining of 8-OHdG in mice lung which was reduced after administration of EGCG (Figure 6A). Nitrotyrosine is a marker of nitrosative stress [21], was significantly increased in the lungs of LPS-instilled mice, treatment with EGCG significantly reduced LPS-induced nitrotyrosine immunostaining in mice lung compared to controls (Figure 6B).

### 2.4. EGCG Administration Regulates Cell Proliferation and Other Inflammatory Markers

**Figure 7:**
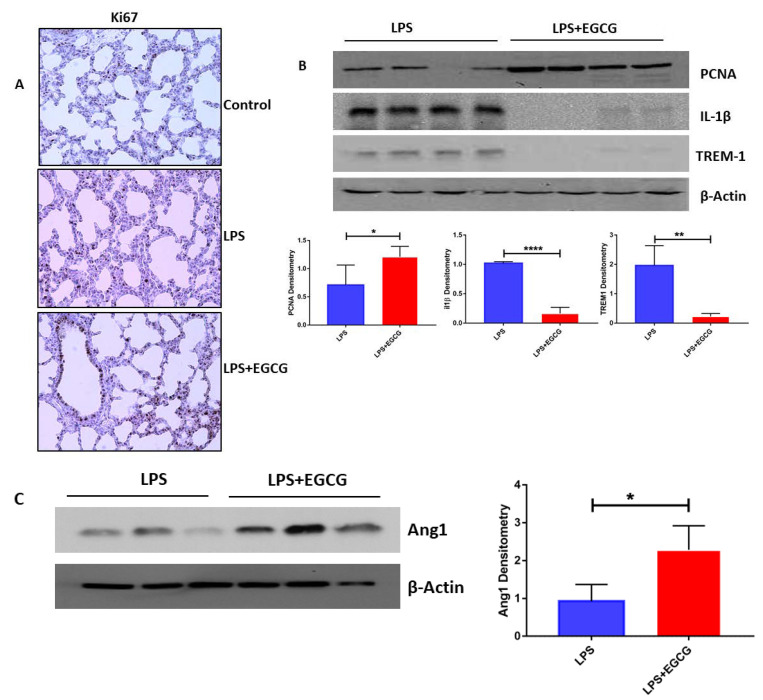
EGCG improves cell proliferation and inhibits other inflammatory mediators. (**A**) The lung Ki67 protein expression shown by immunohistochemistry in the different study groups (10× objective) showed diminished expr of Ki67 in LPS-instilled group, EGCG treatment improved Ki67 staining. (**B**) Western blot of different lung injury groups was performed to evaluate PCNA, TREM1 and IL-1b protein expression (**C**) Immunoblots of Ang1 performed in different lung injury groups. Actin was used in parallel as internal control. Significant difference * *p* < 0.05, ** *p* < 0.01 and **** *p* < 0.0001.

Ki67 and PCNA are markers of cell proliferation and normally expressed within the active dividing cells of bronchial and alveolar lining cells. While in the animals which had undergone LPS-induced ALI, Ki67 was fairly decreased in bronchial and alveolar tissues although EGCG injections improved lung Ki67 immunostaining (Figure 7A). PCNA protein expression was also increased after EGCG injection as compared to the LPS-treated control group (Figure 7B). TREM-1 is a known amplifier of inflammation, specifically expressed in myeloid cells [22,23]. TREM-1 expression was reduced in the LPS + EGCG-treated group as compared to the LPS-alone-treated control group (Figure 7B). Similarly, IL-1β protein expression was also reduced after EGCG treatment in LPS-induced ALI (Figure 7B). Modulation of TREM-1 and IL-1b showed anti-inflammatory potential of EGCG in ALI. Angiopoetin-1 (Ang-1) plays a protective role in ALI and enhanced Ang-1 expression in endothelial or epithelial cells were shown beneficial in different lung injury models [24]. EGCG treatment effectively enhanced Ang1 protein expression in LPS-instilled lung injury model as compared to LPS control (Figure 7C).

## 3. Discussion

Regardless of remarkable developments in medical research, limited discoveries have been made for the treatment of ALI and related disorders, mainly due to the complexity of systemic immune responses [25,26,27]. Accumulating studies proposed that EGCG has many beneficial health effects on different lung ailments [28,29,30]. Therefore, the present study explored EGCG treatment as a probable therapeutic approach for the morphological changes in ALI, which demonstrated that LPS-induced infiltration of inflammatory cells in alveolar spaces, oedema and alveolar damage were improved after concomitant administration of EGCG.

The mice ALI (IT) model exhibited elevated M1 inflammatory factors TNF-α, IL-6 and IL-1β and abrogated M2 markers like Arg1, ym1 etc. Several studies advised that M1 macrophages function as major influencers of sepsis and other ALI [31,32]. The earlier studies showed M1 inflammatory mediators like iNOS, Il-1β, IL-6 etc. are the key players to initiate inflammatory processes and ALI [33,34] and modulation of the M1 inflammatory marker were beneficial for the resolution of acute lung injury [8,34,35,36]. We also showed that lung TNF-α, IL-1β and IL-6 were increased after LPS injection and interestingly concomitant administration of EGCG significantly reduced LPS-induced upregulation of M1 proinflammatory markers. Beneficial effects of EGCG improving inflammatory markers and ALI was already demonstrated by few studies [35,37]. The present study also demonstrated that M2 macrophages were improved after EGCG treatment enhancing several markers like Arg1, ym1 and M2 phenotype regulator KLF4. Enhancement of M2 environment in the lungs has been shown beneficial in acute lung injury and related disorders [8,36]. Several studies also suggested the role of EGCG in enhancing M2 macrophage phenotype in different physiological or pathological conditions [38].

The KLF4 signalling pathway has a key role in the regulation of inflammation, cell proliferation and oxidative stress. KLF4 regulates macrophage polarisation, and has substantial roles in maintaining cellular homeostasis and defending lung injury [12,13]. In the present study, EGCG enhanced the expression of KLF4 in LPS-instilled mice lung. In vitro, RAW264.7 macrophage cells line was used to confirm the effect of EGCG on macrophage polarisation and KLF4 expression. LPS induced a rapid translocation of KLF4 towards the nucleus as the transcription factor and increased the expression of M2 polarisation target genes. KLF4 expression promotes M2 polarisation markers which have demonstrated to regulate the inflammatory response modulating M1 macrophage subset [39]. These points exemplified that the KLF4 signalling pathway is important in modulating macrophage polarisation and inhibition of inflammation [40]. We can assume that EGCG activated KLF4 and polarised macrophages towards the M2 phenotype, would be beneficial in ALI. However, the mechanisms of EGCG-induced KLF4 activation and effect on macrophage polarisation and ALI remain to be determined.

The inflammatory processes and oxidative stress are two major contributors to LPS-induced ALI [41,42,43]. LPS-induced ALI mice models are susceptible to oxidative damage and M2 macrophages are only have a role in regulating the inflammation but also against oxidative and cellular damage [29,44]. 8-OHdG, a marker of oxidative DNA damage was determined in mice lung. The oxidative DNA damage induced by nitrotyrosine (NT) is an appropriate in vivo marker of peroxynitrite [20,21,44]. The development of NO, particularly peroxynitrite, has been implicated in the pathophysiology of several inflammatory diseases, including ALI [44]. We showed EGCG suppressed the expression of inflammatory genes, it also alleviates the production of oxidative stress as demonstrated by 8-OHdG and nitrotyrosine staining. Our results demonstrated less immunostaining of these oxidative stress markers in the EGCG-treated group lungs as compared to the LPS(IT) control group. These may be one of the mechanisms of the defensive function of EGCG in improving LPS-induced ALI. Parallel to our results other studies also stated beneficial effects of EGCG on oxidative damage and ALI [45,46].

Intratracheal instillation of LPS inhibited cell proliferation and enhanced pulmonary cell death [40,47]. Similarly, our study also showed diminished expression of Ki67 and PCNA after LPS instillation in mice lung; however, treatment with EGCG improved the expression of Ki67 and PCNA in lung cells. Ang-1 (angiopoietin-1), an angiogenic marker, demonstrated protective effect in different acute lung injury conditions [24]. We found enhanced expression of Ang-1 after treatment with EGCG in LPS-induced ALI. Similar to this, other studies showed beneficial effects on the regenerative capacity of different lung cells [48,49]. The effect of EGCG might be mediated through KLF4, which is a well−known regulator of stem cells and has a role in cell proliferation [12,50]. TREM-1 is an amplifier of inflammation and expressed in macrophages and other myeloid cells. Heightened expression of TREM-1 was observed in different lung inflammatory conditions and inhibition is beneficial in lung injury [22,51]. Similarly, we also found the suppression of TREM-1 expression in lung macrophages after administration of EGCG in LPS-induced lung injury.

In conclusion, the present study demonstrated that administration of EGCG protected against LPS-induced lung injury in mice models. In addition, EGCG treatment also polarised macrophages toward M2 phenotype regulating KLF4. This study has discovered a strong anti-inflammatory effect of EGCG in LPS(IT) mice model by attenuating lung wet/dry ratio, BAL total leucocyte count, myeloperoxidase and neutrophil infiltration, inflammatory mediators as well as M1 macrophage subsets. EGCG treatment also showed the beneficial impact on LPS-induced oxidative damage and cell proliferation. Further study is required to verify the effect of EGCG in sepsis/infection-induced ALI.

## 4. Methods

### 4.1. LPS-induced ALI Mouse Model

The mice were hosted in compliance with the international guidelines of laboratory animal care, and the procedures on animals were approved by the Institutional Animal Care and Use Committee of College of Applied Medical Sciences, Qassim University (File Number: 1-14-S-3932). Animals were housed in standard cages and during this time, they had free access to food and water. The experimental mice were separated into three groups: (1) PBS-instilled control, (2) LPS-instilled (2 mg/kg body weight) and (3) LPS + EGCG (15 mg/kg). The 15 mg/kg EGCG was administered IP injection for twice and 50 μL of LPS (2 mg/kg) or PBS were-instilled intratracheally to induce acute lung injury. The mice were sacrificed 18 h after the LPS treatment using ketamine/xylazine, and then the bronchoalveolar lavage fluid (BALF) was harvested using chilled PBS (10 mL.X3). The BAL fluid was centrifuged, and one part of cells was stained with Trypan blue and counting was done using a haemocytometer to determine the total leukocyte count. Remaining cell pellet resuspended again with PBS and cytospin centrifuge was used to prepare a single layer of BALF cells, which were stained with HEMA stain (Thermo Fisher Scientific, Waltham, MA, USA). The supernatant was used to perform cytokines and MPO quantifications. The lung tissue was fixed in 4% neutral buffered formalin (Sigma-Aldrich, MO, USA), embedded in paraffin, and performed sectioning using microtome [44].

### 4.2. Lung Wet to Dry Weight Ratio

After completion of the experiment, a portion of the right lung of each mouse was removed and weighed. Next drying of the same tissue at 60 °C for 48 h in oven, then it was reweighed. Lung tissue oedema was calculated using wet/dry weight ratio measurements [52].

### 4.3. Immunofluorescence Staining

RAW264.7 cells were plated on chambered slides, then treated with EGCG and LPS for the specified time period. After treatment, macrophages were fixed with 4% paraformaldehyde for 30 min and then permeabilisation was performed with 0.1% Triton X-100 for 5 min. The cells were blocked with BSA and incubated with the KLF4 antibody (1:50) for 2 h at room temperature. Then, the cells were incubated with the Alexa Fluor 488-conjugated secondary antibody (Jackson Immuno-research) for 1 h at room temperature. Counterstaining was done with DAPI (Invitrogen, USA) and observed by a fluorescence microscope.

### 4.4. Histopathological Analysis

Lung tissues were collected and fixed in 10% neutral buffered formalin (Sigma) for 48 h. The lung tissue was embedded in paraffin wax, sectioned into 5 µm slices and stained with haematoxylin and eosin (H&E). Images of sections were analysed under a light microscope, captured and results were interpreted accordingly [22].

### 4.5. Immunohistochemical Staining

For IHC, these tissue sections were deparaffinised and rehydrated followed by heat-induced epitope retrieval for 20 min. The lung tissue sections were blocked for 1 h and then incubated with primary antibodies against KLF4, Ki67, 8-OHDG, and nitrotyrosine (Santacruz Biotechnology, Santa Cruz, CA, USA). For further procedure, ABC staining kits (Vector) were used to stain all tissues. Finally, diaminobenzidine (DAB), chromogen was used, the section was counterstained with haematoxylin and results were interpreted under a light microscope and a photograph was taken.

### 4.6. Immunoblotting Analysis

RAW264.7 macrophages or lung tissues of different treatment groups. The cells/tissues were lysed with RIPA lysis buffer (Santacruz Biotechnology, Santa Cruz, CA, USA). The proteins were quantified and equally loaded for SDS–PAGE, and after completing the run, PVDF membrane was used to transfer the proteins. The membranes were then blocked with 5% milk (Bio-Rad, Hercules, CA, USA) at room temperature for 1 h. The membranes were then incubated overnight with KLF4, Ang1, iNOS, Cox-2, PCNA, ym1 arg1 and il-1β antibodies (1:1000 in 4% milk, Bio-Rad) at 4 °C overnight and then incubated with the secondary antibodies (1:5000) for 1 h. The bands were visualised with ECL (Bio-Rad). β-actin was used as internal controls.

### 4.7. Semi-Quantitative RT-PCR

RNA was isolated from RAW macrophages using RNeasy kit (Qiagen, USA). Total RNA concentration was determined using spectrophotometer and reverse-transcribed into cDNA with iScript cDNA Synthesis Kit (Bio-Rad). RT-PCR was performed with 1 μL of cDNA in PCR System (Bio-Rad). The primers used were: 5′ -GAGCGAGTTGTGGATTGTC-3′ (F) and 5′-CTCCTTTGAGCCCTTTGT-3′ (R) for iNOS; and 5′ -CTGTCCCTGTATGCCTCTG-3′ (F) and 5′ -ATGTCACGCACGATTTCC-3′ (R) for β-actin. The amplified genes were subjected to 1.2% agarose gel electrophoresis.

### 4.8. Myeloperoxidase Assay

Lung tissues were placed in phosphate buffer (pH 6.0) containing 0.5% hexadecyl trimethyl ammonium bromide and stored at −80 °C until assayed. A piece of lung from each animal was homogenised in the same buffer and centrifuged at 12,000 rpm for 30–40 min at 4 °C. *o*-Dianisidine dihydrochloride in 0.0005% hydrogen peroxide in phosphate buffer was then added to 10 μL supernatant in a cuvette. Change in absorbance was determined at 460 nm for 3 min. Protein concentrations were determined by Bio-Rad protein assay kit. Myeloperoxidase (MPO) activity was expressed as change in absorbance per minute per mg of protein [21].

### 4.9. Cytokines Determination

BAL fluid was used to determine cytokine concentration. Protein concentration was determined with the Bradford Protein Assay kit (Bio-Rad). TNFα and IL-6 concentrations were measured with an ELISA kit (R&D) as instructed.

### 4.10. Statistical Analyses

All results are provided as the means ± SEM. Statistical assessments were done using one-way or two-way ANOVA. Significance was demarcated as *p* *<* 0.05.
